# Supplementary material for: Learning curve and surgical outcome of robotic assisted colorectal surgery with ERAS program
Source: Sci Rep. 2022 Nov 29;12:20566. doi: 10.1038/s41598-022-24665-w (PMC9709162; doi:10.1038/s41598-022-24665-w)
Supplement: Supplementary file 2 — Supplementary Information 2. [file 41598_2022_24665_MOESM2_ESM.zip › RAL Raw data-Ñ[▒K/103/HS_ñw╝╨░O▒Kñσ.pdf]

正式摘要(電子簽章：000000223819923)<br>W52 / W52 ☐ 英文標題(注意:無英文姓名-請補病人基本檔!)	☐【讀取】憑證

臺中榮民總醫院

出院病歷摘要

轉入號： 轉出號：

[redacted]

(8)流水編號	
(9)入院日期	110/08/04 (2021) 直腸外科 W52 - 145
(10)轉科日期	
(11)出院日期	110/08/26 (2021) 住院天數計 22 日

診斷

- 入院：1. Rectal adenocarcinoma, at 7cm FAV, cT2N0M0, s/p Robotic LAR on 2021/08/05
  -/c minor anastomosis leakage s/p ileostomy on 2021/08/09
- 出院：1. Rectal adenocarcinoma, at 7cm FAV, cT2N0M0, s/p Robotic LAR on 2021/08/05
  -/c minor anastomosis leakage s/p ileostomy on 2021/08/09
  2. Acute cholecystitis, s/p conservative Tx (2021/08/17-08/24)

主訴

- mild pitting edema over biltaeral lower limbs for one year

病史

- This 80-year-old man denied underlying disease complained about mild pitting edema over biltaeral lower limbs for one year. He had initial medical visit to local clinic, mild anemia and stool occult blood positive were noted. He came to our hospital and colonscopy showed one rectal tumor 3-4cm in size, at 7cm FAV, pathology showed adenocarcinoma, moderately differentiated. As above problems, he was admitted for surgical treatment of robotic low anterior resection.

體檢發現

-
  - General appearance :
    - easy looking , conscious clear , without cardiopulmonary distress
    - Vital signs自動帶入date 體溫(BT) 脈搏(P) 呼吸(R) 血壓(BP) 身高(BH) 體重(BW) BMI
      20210804 36.5℃ 61次/分 18次/分 156/61mmHg 162.5cm 64.2kg 24.3
    - Vital signs: B.P. 156/61 mmHg , P.R. 61 /min , R.R. 18 /min , B.T. 36.5 ℃
  - HEENT :
    - Eyes: no anemic conjunctiva, no icteric sclera, normal EOM
    - ENT: no otorrhea, no rhinorrhea, no erythematous mucosa, no injected tonsil, no deformity
  - Neck: supple, freely movable, trachea midline,no deformity, no engorged jugular vein , no palpable thyroid or lymph node enlargement
  - Chest:
    - Inspection: Grossly normal, symmetric and fully expansion, no accessory muscle used, no operation scar, no spider angiomata, no gynecomastia
    - Palpation: no palpable mass or nodule, no subcutaneous emphysema, no tenderness
    - Ausculation: clear breathing sound
  - Heart:
    - Percussion: normal heart size
    - Ausculation: regular heart beat, normal S1, S2, no gallop, no murmur
  - Abdomen:
    - Inspection: in
      - flat contour, symetrically, no scar, no stria, no dilated veins, no rashes
    - Ausculation: normoactive bowel sound, no abdominal bruits
    - palpation: soft, no muscle guarding or rigidity, no local tenderness, no rebounding pain, no palpable mass, no palpable pulsation
    - Percussion: no shifting dulllness, no hepatomegaly, no splenomagaly
  -

Peripheral pulsation :	CA	BA	RA	FA	PA	PTA	DPA
Right side :	++	++	++	++	++	++	++/+++
Left side :	++	++	++	++	++	++	++/+++

  - Genito-anus: no tenderness or palpable mass lesion or rectal shelf, normal external genitalia, normal anal tone, brown stool

CRS CONDITION

- CEA: 2.89 ng/ml
- Imaging studies(Sono , CT of abdomen and chest Films):
  - 檢驗項目 :CT ABDOMEN-SURVEY 報告日期 :1100719
    主 訴 :
    Result:Y Finding: Imaging Report Form for Colorectal Carcinoma (AJCC 8th) 1. Imaging Date: 20210715 Imaging Modality, Imaging by CT scan Imaging Protocol: CT scan of abdomen with IV and oral contrast enhancement: 2. Tumor location / Size Location: Rectum Size: Non-measurable 3. Tumor invasion Tumor cannot be visualized in this imaging study 4. Regional nodal metastasis No 5. Distant metastasis (In this study) No or Equivocal 6. Other findings: 1. A case of R/O rectal cancer for survey. 2. The primary lesion of rectum can not be detected within this study. 3. Clips at cecum & D-colon, may be related to post-procedure change. 4. Moderate fatty liver 5. Small bilateral renal cysts. 6. gallbladder, pancreas & spleen are without specific abnormality. 7. No obvious para-aortic or retrocaval lymphadenopathy is noted. 8. A ground glass nodule about 0.5cm in size at LLL(ax.7). Suggest regular F/U. 9. DJD changes over T-L spine Impression: 1. The primary lesion of rectum can not be detected within this study. 2. a ground glass nodule at LLL. Suggest regular F/U. 3. Other findings, as above description
-
  - Lung

手術日期及方法

- 手術日期及方法20210809 Loop ileostomy
  20210805 1. Robotic (XI) assisted low anterior resection
  2. Intraoperative colonoscopy : (-).
  3. High ligation (+) : At IMA root (Via endoclips). IMV was also ligated via endoclip.
  4. Take down from the splenic flexure : (-)
  5. Robotic endoGIA (60-4.3) X2 + EEA 33 mm (Echelon circular powered stapler) to complete end-to-end anastomosis.
  6. Proximal cut end: 11cm, distal cut end: 2 cm
  7. No drainage tube was inserted
  8. ADHESION BARRIER FILM was placed below the extended wound
  9. Loop ileostomy (About 15~20cm from IC valve) was done over RLQ of abdomen
- 手術結果及發現：
  - 1. A 2.5*3 cm Borrmann type III tumor at lower rectum, which invaded to the muscular layer, but didn't penetrate through the serosa. One enlarged lymph nodes at mesorectum, cT2-3N1M0. 3. The previous suture from per-anal exicision was clearly seen. 4. No other gross tumor nor polyps was found 5. There was no obvious visible liver metastasis, no visible peritoneal seeding 6. No obvious ascites was noted 7. Bilateral doughnuts : intact ; Air leak test: Done and no air leak was found

住院治療經過

- He underwent robotic-assisted LAR on 2021/08/05. After surgery, he had regained conscious and tolerated well under PCA control. We kept ERAS-based protocol for postoperative care. He had smooth oral intake and bowel gas passage at POD-1. However, he was suffered from intermittent fever since 8/7, he also complained about abdominal fullness and mild N/V with chest burning sensation. PE showed distented abdomen, tympanic percussion, no abdominal tenderness nor muscle gaurding. Follow-up CXR showed increasing subprenic free air at right side. Lab data showed high lactate and CRP level. Abdominal CT showed pneumoperitonium and diffuse fat stranding over mesentey and mild ascites. As the suspicous of anastomosis leakage, we performed emergent exploratoy laparotomy on 08/09, there was no visible intestine rupture nor anastomosis rupture but some turbid ascites accumulation at pelvic wall, and eventually we performed ileostomy for stool diversion. We shifted Abx to Tazocin for seven days (08/09-08/16). After re-do surgery, he presented afebrile and had much improvement on distented abdomen. He started to

- try water on 08/11, then liquid and semi-liquid diet smoothly. Much fluid discharge from ileostoma was noted, we kept adequate IV repletion and self-paid Albumin supplment. He had fair oral intake of semi-liquid diet and shifted to soft diet on 08/17, some loose stool from ileostoma was noted. However, he was suffered from persistent low grade fever without tachycardia since 08/17, he denied cough, thick sputum, abdominal pain, dysuria, while PE showed mild Murphy's sign, lab data showed leukocytosis and mild elevation of bilirubin level. Abdominal echo(8/18) showed gallbladder sludge and R/I acute cholecystitis. We kept empirical antibiotic with Tazocin since 8/17-08/24. Fever had subsided and follow up lab data showed improvement. Under his relative stable condition, he was discharged on 08/26 and arranged OPD f/u.

合併症

- 1. -/c minor anastomosis leakage s/p ileostomy on 2021/08/09 2. Acute cholecystitis

一般檢查

- 累積報告：

DATE	TIME	ALB	TP	BIL.T	BIL.D	ALKP	AST	ALT	LDH	AMY	LIPASE	NH3	Ferritin	UA
1100804	1704	4.1		1.30	0.26		24	24						
1100805	2027	3.4		1.1			18	20						
1100807	1508			1.45		62	20	15						
1100809	1127	2.9		1.32	0.39		25	12	212					
1100814	0902	2.9		1.33	0.51									
1100817	0733			1.47	0.45	114	23	21						
1100819	0849	2.8		0.97	0.33									
1100823	0855			0.77	0.27			12						

DATE	TIME	WBC	RBC	HGB	HCT	MCV	PLT	BLST	NEUT	BAND	SEG	LYM	MON	EOS	BAS
1100804	1704	7490	6.36	12.5	40.3	63.4	218		61.7			29.4	6.5	1.9	0.5
1100805	2026	13170		12.3			244		91.7			6.1	2.1	0.0	0.1
1100807	1505	11760		11.7			172		89.3			6.6	3.8	0.1	0.2
1100809	1128	10520	5.90	11.7	35.9	60.8	160			5.0	84.0	9.0	1.0	1.0	0.0
1100811	0852	9520		10.1	30.9		159		83.3			6.8	5.4	4.3	0.2
1100814	0906	9300		10.3			261		72.9			15.3	8.0	3.5	0.3
1100817	1654	16990		11.3	35.2		523		87.2			6.7	5.6	0.2	0.3
1100819	0913	12430	4.86	9.5	28.8	59.3	475		81.3			9.7	5.9	2.8	0.3
1100823	0925	12130		10.0	30.6		521		78.4			12.0	6.5	2.4	0.7
1100825	0711	9900		10.0	32.2		507		73.9			14.2	7.2	3.8	0.9

DATE	TIME	NA	K	CL	BUN	CREAT	eGFR	CA	Mg	P	CRP	ESR	PCT	D-Dimer	LACTATE
1100804	1704	141	4.3		19	0.86		8.6							
1100805	2027	141	3.8		10	0.72									
1100807	1506														10.0
1100807	1508	138	3.3		6	0.78	101.79								
1100809	1115														14.2
1100809	1127	134	4.0		14	0.79		7.2			28.205				
1100811	0855	140	3.2		16	0.82	96.08						6.480		
1100814	0902	139	3.9					7.2			4.582				
1100817	0733	134	3.8			0.75	106.5						0.439		
1100817	1654														13.2
1100819	0648														6.1
1100819	0849	135	4.3					7.5			11.348				
1100823	0734														6.1
1100823	0855	136	4.0		8	0.70	115.33	7.5			3.203				
1100825	0712	137	4.8					8.0			4.317				

DATE	TIME	SP-GR	PH	PROT	GLUC	KETON	BILI	UROB	OB	NITR	LEU	RBC	WBC	EPCEL
1100807	1525	1.014	5.5	Negative	Negative	Negative	Negative	0.2	Negative	Negative	Negative	0-2	5-10	0-1
1100809	1715	1.027	6.5	2+	Negative	Trace	1+	0.2	Trace	Negative	Trace	0-2	2-5	0-1
1100817	1046	1.019	5.5	Trace	Negative	Negative	Negative	0.2	Negative	Negative	Negative	0-2	0-2	0-1

DATE	TIME	PH	BEB	SCVO2	PO2	HCO3	HGB	PCO2	TCO2
1100807	1513	7.359	3.0	60.7	32.9	29.7		54.0	31.4
1100809	1122	7.430	3.1	71.7	36.2	27.9		43.0	29.2
1100811	0803	7.427	3.4	93.4	65.8	28.4		44.1	29.7
1100817	1654	7.426	1.5	34.8	20.6	25.9		40.3	27.2
1100819	0653	7.422	0.2	94.3	70.4	24.4		38.3	25.6

- 放射線報告：檢驗項目 **:SONO,ABDOMEN** 報告日期 **:1100818**

主 訴：
Result:N
Finding:
1. Moderate fatty liver
2. Gall bladder sludge and slight gall bladder distension, acute cholecystitis cannot be ruled out.
Suggest further evaluation.
3. A 1.5cm renal cyst in right kidney
4. Right kidney is 12.1cm. Left kidney is 12.1cm. No hydronephrosis.
5. Mild ascites is noted.
6. Pancreas and spleen are not unusual.
Impression:
1. Moderate fatty liver
2. Gall bladder sludge and R/I acute cholecystitis
3. Right renal cyst
4. Mild ascites

檢驗項目 **:ABDOMEN (KUB),SUPINE** 報告日期 **:1100823**
主 訴：
Result:N
Finding:
> Some gaseous bowel loop dilatation in the abdomen, ileus is considered and obstruction cannot be ruled out.
> DJD change of L-spine with marginal spur formation.
> S/P ostomy over RLQ area with drainage tube insertion
> Surgical autosuture in pelvic region
Impression:
As above description.

特殊檢查

病理報告

- 活體組織切片病理報告 手術日期：**110/08/05** 病理號：**S1024079**

診斷醫師:胡仔璇 報告日期:110/08/13
Pathologic diagnosis:
1. Rectum, robot low anterior resection --- Adenocarcinoma, moderately differentiated. Tumor invaded to perirectal soft tissue, and metastasized to perirectal lymph nodes (6/17). The bilateral cut ends are free of tumor.
2. IMA root, dissection --- Unremarkable fibroadipovascular tissue.
3. Colon, cut end, distal, resection --- Unremarkable.
Ancillary study for diagnosis:
IHC stains for PMS2 and MSH6 are done on section H. Both above markers are preserved.
Prognostic and predictive factor:
1. Tumor size: 3.5 cm.
2. Depth of invasion: Perirectal soft tissue.
3. Histologic grade: G2: Moderately differentiated.
4. Angiolymphatic invasion: Not identified.
5. Perineural invasion: Present.
6. Tumor deposits (discontinuous extramural extension): Not

- identified.
  7. Macroscopic tumor perforation: Not identified.
  8. Surgical Margins: a) Proximal Margin: Uninvolved by invasive carcinoma, distance of tumor from margin: 9 cm. b) Distal Margin: Uninvolved by invasive carcinoma, distance of tumor from margin: 2.6 cm. c) Radial or Mesenteric Margin: Uninvolved by invasive carcinoma, distance of tumor from margin: 1.5 cm.
  9. Lymph node status: Involved (6/17).
  10. Extranodal involvement: Present.
  11. Tumor regression grading S/P CCRT: No known presurgical therapy.
  12. Type of polyp in which invasive carcinoma arose: Not identified.
  13. Additional pathologic findings: Not identified.
  14. Pathological TNM Stage: pT3N2a(According to the eighth edition, American Joint Committee on Cancer Staging Guidelines for Tumors).
  15. TNM descriptors: Not applicable.
  Gross description:
  The specimen consists of 1) a segment of colon, 15 cm in length and 6.5 cm in proximal cut end circumference, 5 cm in distal circumference, and perirectal soft tissue. A gray tan firm annular elevated and centrally ulcerative tumor, 3.5x3.2 cm, is noted, 2.6 cm to the nearest cut end. On cross section, the tumor invades to the perirectal soft tissue with 15 mm to the radial margin. The both cut ends are free of tumor grossly. 2) IMA root, 2 pieces of tan yellow soft tissue, up to 4.5x2.2x0.5 cm. 3) distal cut end, a piece of tan mucosa-covered soft tissue, 1.5x1.4x1 cm. Representative part for section: A) specimen 2 B) specimen 3 all for section C) proximal cut end D) distal cut end E) tumor and CFM F-H) tumor J-L) lymph nodes.
  Note:
  1. The interpretation of MMR panel and recommended studies are as below: (1) No loss of nuclear expression of MMR proteins: low probability of microsatellite instability-high (MSI-H) . (2) Loss of nuclear expression of MLH1 and PMS2: testing for methylation of the MLH1 promoter and/or mutation of BRAF is indicated (the presence of a BRAF V600E mutation and/or MLH1 methylation suggests that the tumor is sporadic and germline evaluation is probably not indicated;
  absence of both MLH1 methylation and of BRAF V600E mutation suggests the possibility of Lynch syndrome, and sequencing and/or large deletion/duplication testing of germline MLH1 may be indicated). (3) Loss of nuclear expression of MSH2 and MSH6: high probability of Lynch syndrome (sequencing and/or large deletion/duplication testing of germline MSH2 may be indicated, and, if negative, sequencing and/or large deletion/duplication testing of germline MSH6 may be indicated). (4) Loss of nuclear expression of MSH6 only: high probability of Lynch syndrome (sequencing and/or large deletion/duplication testing of germline MSH6 may be indicated). (5) Loss of nuclear expression of PMS2 only: high probability of Lynch syndrome (sequencing and/or large deletion/duplication testing of germline PMS2 may be indicated). (6) Please note: There are exceptions to the above IHC interpretations. These results should not be considered in isolation, and clinical correlation with genetic counseling is recommended to assess the need for germline testing.
  2. This case has been peer reviewed by two doctors.
  #T-59300_2 #M-81403_2 2 1105 S0000F
  #T-C4560_2 #M-81406_2 2 1105 000000

其它

出院時情況

- 3.改門診治療

出院指示

藥名	劑量	劑量單位	用法	頻次	數量	數量單位	現況	天數	單位
Econ tab 40mg	1	TAB	PO	QID	24	TAB	TH	6	天
Ulstop tab 20mg	1	TAB	PO	BID	12	TAB	TH	6	天
$Miyarisan BM powd.	1	PACK	PO	TID	18	PACK	TH	6	天

出院計劃

- 門診日期
  - date on 2021/08/31
- 衛教：一、飲食攝取：1.少量多餐。2.禁食刺激性食物，如太辣、太膩，大量飲酒及抽太多煙、濃茶和咖啡。3.多攝取高蛋白食物，以加速傷口修復。如：新鮮瘦肉、魚、蛋等。4.並減少吃產氣食物可避免脹氣。如：牛奶、豆類、地瓜、蛋、洋蔥。二、術後活動：1.三個月內不可從事下列活動：騎馬、騎腳踏車、劇烈的跳舞、久坐及開車，以免骨盆充血。2.手術後６？８週內避免提超過5KG之重物，若提物品可使用腹托或束腹帶以免震動所引起之疼痛。3.依個人的體力採漸進增加活動量，可先採輕工作，如無不適，體力許可再增加活動量。三、傷口護理：1.保持傷口清潔、乾燥，若有不慎弄濕傷口，可用清潔的毛巾或紗布拭乾。2.每日觀察傷口，是否有紅、腫、熱、痛，或不正常的分泌物。四、居家服藥：1.帶回的藥物，注意藥袋上的服用劑量、時間及注意事項。2.服用軟便劑若解便正常或有腹瀉現象時應停藥或減量。 五、出院後，若有下列情況需立即返院檢查：1.高燒不退。2.腹部疼痛厲害或嚴重腹脹嘔吐。3.傷口流血不止，或是有不正常之分泌物4.有其它問題，請與大腸直腸外科相關人員聯絡 TEL: 04-23592525 轉5161-3 或 6051, 6052
- OPD f/u

主治醫師 2062F 蔣鋒帆 / 住院醫師 2336L 洪浚嚴

正式摘要(電子簽章：000000223819923)

W52 / W52

doctorRank:B
